# Supplementary material for: How fast are viruses spreading in the wild?
Source: PLoS Biol. 2024 Dec 3;22(12):e3002914. doi: 10.1371/journal.pbio.3002914 (PMC11614233; doi:10.1371/journal.pbio.3002914)
Supplement: S1 Text — (PDF) [file pbio.3002914.s001.pdf]

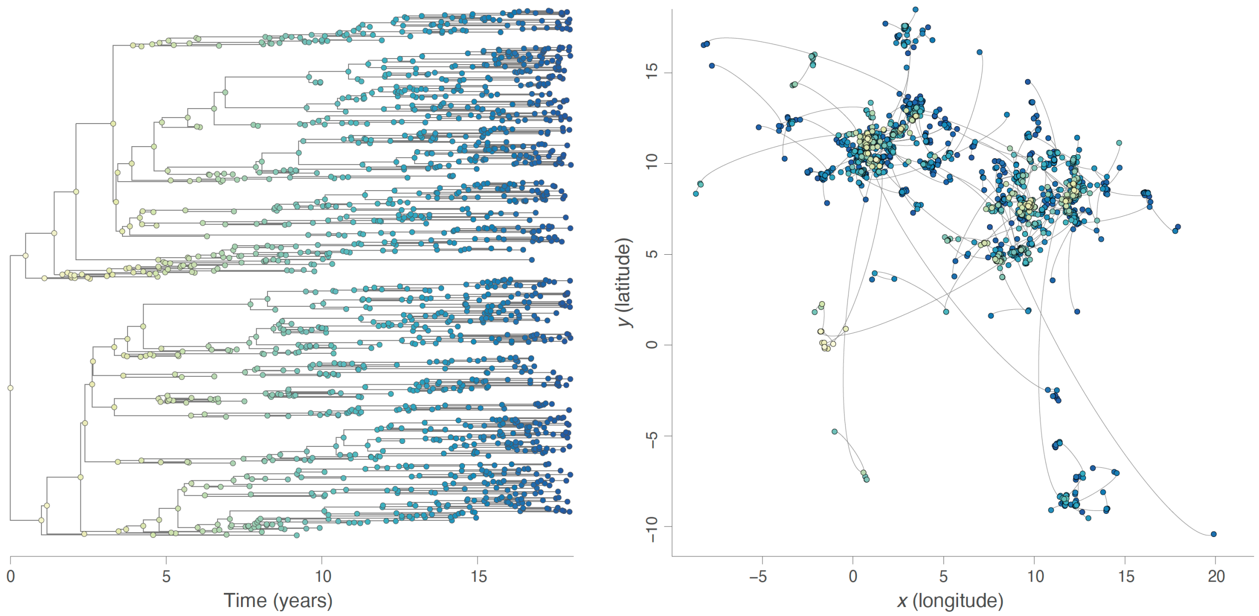

Figure A: example of a continuous phylogeographic simulation based on a relaxed random walk (RRW) diffusion process with phylogenetic trees simulated under a birth-death model. Both graphs display the phylogenetic tree sampled during a unique simulation, with its time-scaled visualisation in the left panel and its mapped visualisation in the right panel. Tree nodes are coloured according to time, with internal and tip nodes coloured according to their time of occurrence and collection time, respectively. The data underlying this figure can be found in https://doi.org/10.5281/zenodo.13984927.

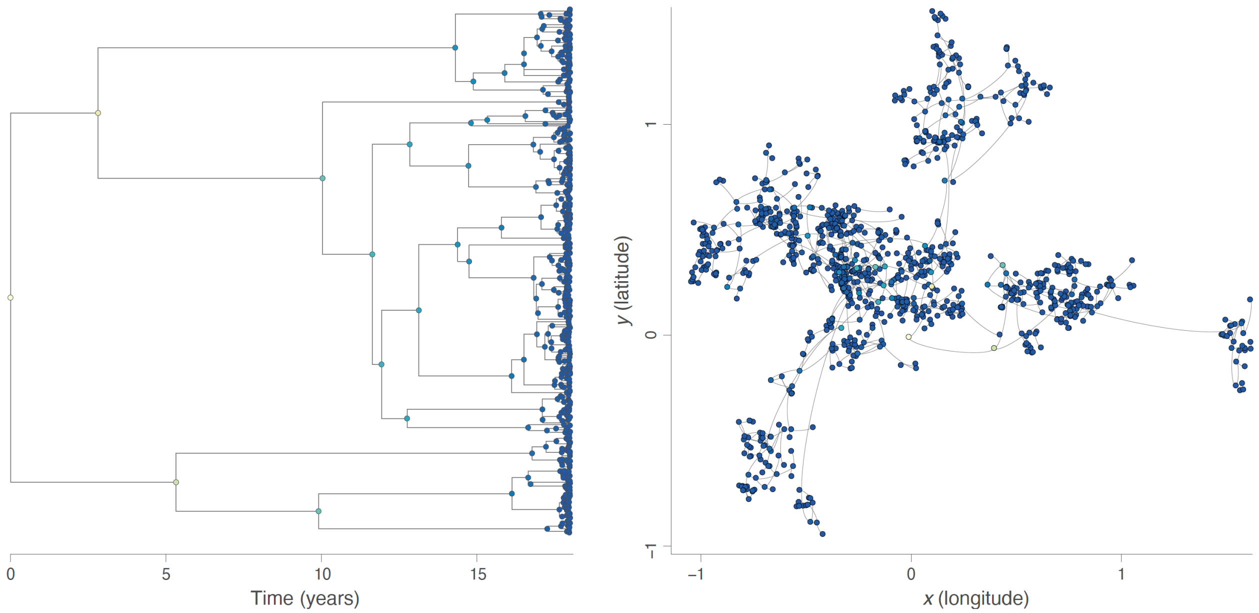

Figure B: example of a continuous phylogeographic simulation based on a relaxed random walk (RRW) diffusion process with phylogenetic trees simulated under a coalescent model. Both graphs display the phylogenetic tree sampled during a unique simulation, with its time-scaled visualisation in the left panel and its mapped visualisation in the right panel. Tree nodes are coloured according to time, with internal and tip nodes coloured according to their time of occurrence and collection time, respectively. The data underlying this figure can be found in https://doi.org/10.5281/zenodo.13984927.

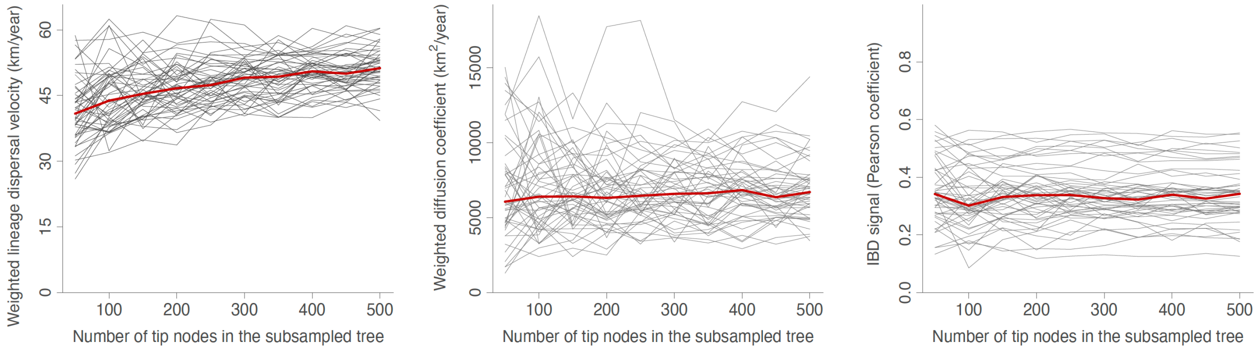

Figure C: robustness of lineage dispersal metrics to the sampling intensity, here based on the relaxed random walk (RRW) simulations where phylogenetic trees were simulated under a birth-death model. In complement to Fig 1 reporting the corresponding results based on Brownian random walk (BRW) simulations, we here report the same dispersal statistics estimated on 50 geo-referenced phylogenetic trees simulated under a RRW diffusion process. Each specific tree is represented by a specific grey curve obtained when re-estimating the dispersal metric on subsampled versions of the tree, i.e. subsampled trees obtained when only randomly keeping 500, 450, 400, 350, 300, 250, 200, 150, 100, and 50 tip nodes; and the red curve indicate the median value across all simulated trees. The data underlying this figure can be found in https://doi.org/10.5281/zenodo.13984927.

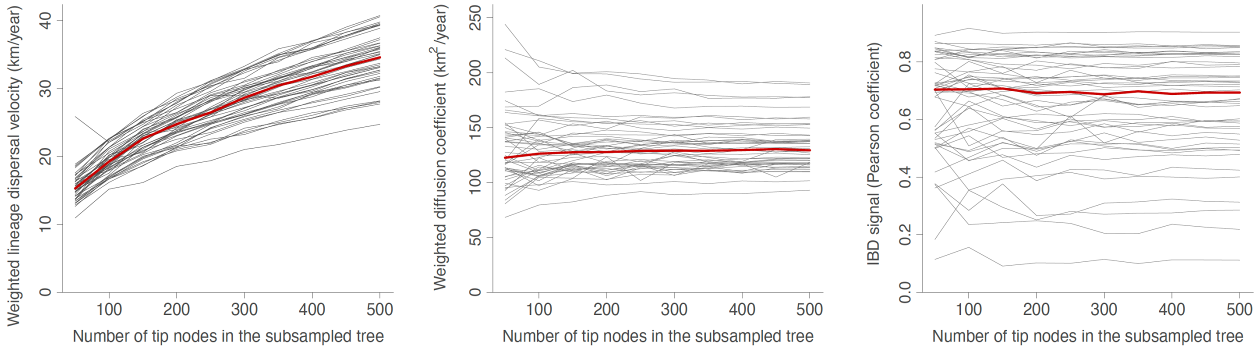

Figure D: robustness of lineage dispersal metrics to the sampling intensity, here based on the Brownian random walk (BRW) simulations where phylogenetic trees were simulated under a coalescent model. In complement to Fig 1 reporting the corresponding results from BRW simulations where phylogenetic trees were simulated under a birth-death model, we here report the same dispersal statistics estimated on 50 geo-referenced phylogenetic trees simulated under a BRW diffusion process this time based on tree topologies simulated under a coalescent model. Each specific tree is represented by a specific grey curve obtained when re-estimating the dispersal metric on subsampled versions of the tree, i.e. subsampled trees obtained when only randomly keeping 500, 450, 400, 350, 300, 250, 200, 150, 100, and 50 tip nodes; the red curve indicating the median value. The data underlying this figure can be found in https://doi.org/10.5281/zenodo.13984927.

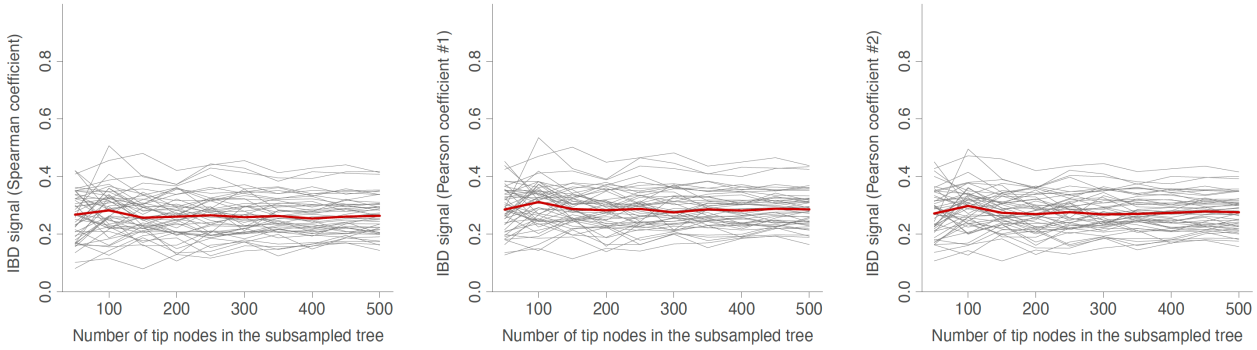

Figure E: robustness of alternative isolation-by-distance (IBD) metrics to the sampling intensity, here based on the Brownian random walk (BRW) simulations where phylogenetic trees were simulated under a birth-death model. In complement to Fig 1, we here report three distinct iBD signal metrics estimated on the same 50 geo-referenced phylogenetic: (i) the Spearman correlation coefficient between the patristic and great-circle geographic distances computed for each pair of tip nodes, (ii) the Pearson correlation coefficient (#1) between the patristic and great-circle geographic distances computed for each pair of tip nodes, and (ii) the Pearson correlation coefficient (#2) between the patristic and log-transformed great-circle geographic distances computed for each pair of tip nodes. Each specific tree is represented by a specific grey curve obtained when re-estimating the dispersal metric on subsampled versions of the tree, i.e. subsampled trees obtained when only randomly keeping 500, 450, 400, 350, 300, 250, 200, 150, 100, and 50 tip nodes; and the red curve indicate the median values. The data underlying this figure can be found in https://doi.org/10.5281/zenodo.13984927.

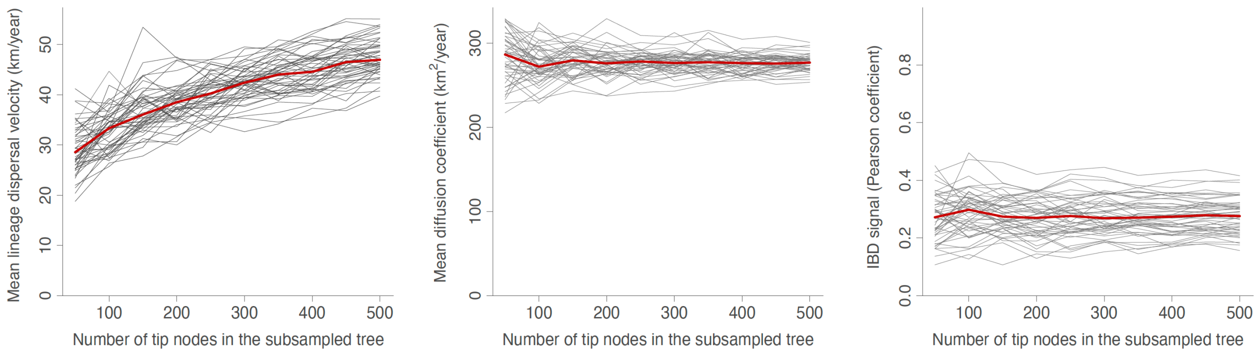

Figure F: robustness of alternative dispersal metrics to the sampling intensity, here based on the Brownian random walk (BRW) simulations where phylogenetic trees were simulated under a birth-death model. In complement to Fig 1 reporting the results obtained for the weighted lineage dispersal velocity (WLDV, km/year) and weighted diffusion coefficient (WDC, km^2/year), we here report the mean lineage dispersal velocity (MLDV, km/year) and mean diffusion coefficient (MDC, km^2/year) estimates. As in Fig 1, we also report estimates for the isolation-by-distance (IBD) signal has been estimated by the Pearson correlation coefficient between the patristic and log-transformed great-circle geographic distances computed for each pair of tip nodes. Each of the 50 simulated trees is represented by a specific grey curve obtained when re-estimating the dispersal metric on subsampled versions of the tree, i.e. subsampled trees obtained when only randomly keeping 500, 450, 400, 350, 300, 250, 200, 150, 100, and 50 tip nodes; and the red curve indicate the median value across all simulated trees. The data underlying this figure can be found in https://doi.org/10.5281/zenodo. 13984927.

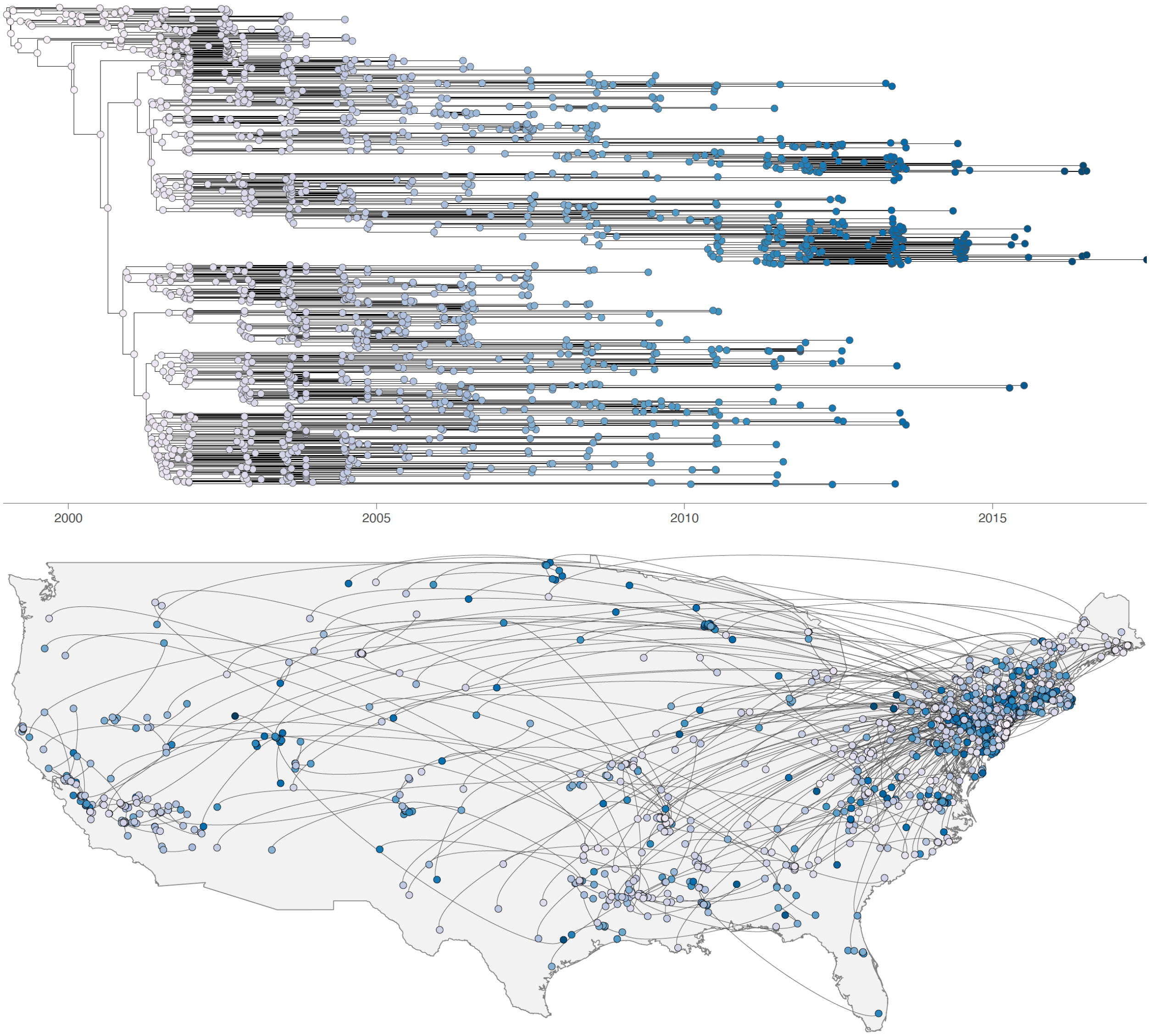

Figure G: example of a continuous phylogeographic simulation based on a relaxed random walk (RRW) diffusion process simulated along the branches of the maximum clade credibility (MCC) tree obtained from the continuous phylogeographic reconstruction itself based on West Nile virus genomes collected between 1999 and 2016 in North America [1]. Both graphs display the phylogenetic tree resulting from a single simulation, with its time-scaled tree visualisation in the top panel and its mapped visualisation in the bottom panel. Tree nodes are coloured according to time, with internal and tip nodes coloured according to their time of occurrence and collection time, respectively. Source of the administrative shapefile used to generate the map: Database of Global Administrative Areas (GADM, gadm.org). The data underlying this figure can be found in https://doi.org/10.5281/zenodo.13984927.

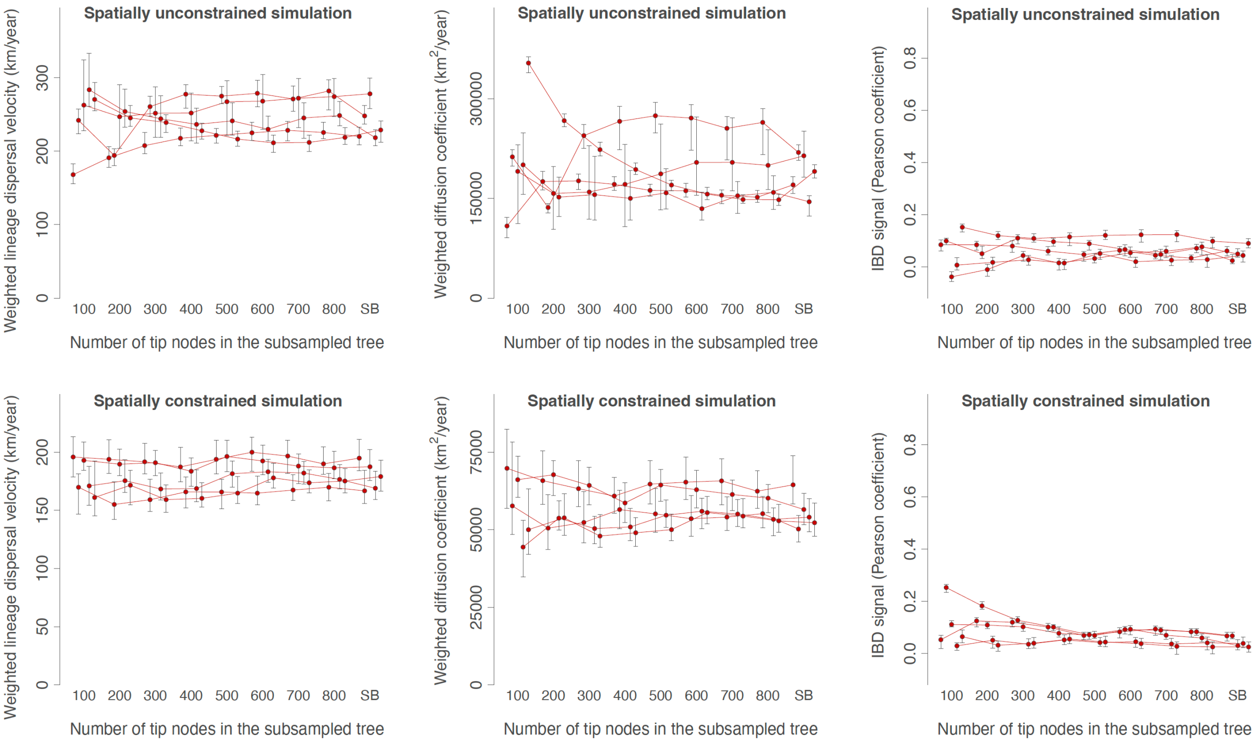

Figure H: robustness of lineage dispersal metrics to the sampling intensity and bias while incorporating the uncertainty associated with the phylogenetic inference. We report the three dispersal statistics under investigation — the weighted lineage dispersal velocity (WLDV), the weighted diffusion coefficient (WDC), and the isolation-by-distance signal (r_P) metrics — estimated from a set of posterior trees each time sampled from a continuous phylogeographic inference based on datasets simulated for the West Nile virus spread in North America [1] (see the text for further detail on the simulation process illustrated in Figure G in S1 Text). In the different graphs, the vertical intervals correspond to the 95% highest posterior density (HPD) interval of dispersal metrics estimated on 100 posterior trees, and the red dots to the corresponding median estimates. Simulations were either spatially unconstrained (first row of graphs) or constrained (second row of graphs) to the mainland United States (excluding Alaska). For each of these two categories of simulations, we first considered datasets resulting from various sampling intensities, ranging from 100 to 800 sampled genomes randomly sampled within the original West Nile virus dataset. Secondly, for each category of simulations, we also considered a scenario of sampling bias (SB), which corresponded to a sampling restricted to all genomes sampled east of the tree root location for the unconstrained simulations (corresponding, on average, to half of the simulated samples), and all genomes further east of the 86th West meridian for the spatially constrained simulations. In the latter case, with a root location inferred in the New York city area for the West Nile virus invasion in North America, it roughly corresponds to a subsampling restricted to the first eastern third of the invaded area across mainland USA. In the different panels, the red line segments link the results obtained for a distinct initial RRW simulation conducted along the MCC tree. The data underlying this figure can be found in https://doi.org/10.5281/zenodo.13984927.

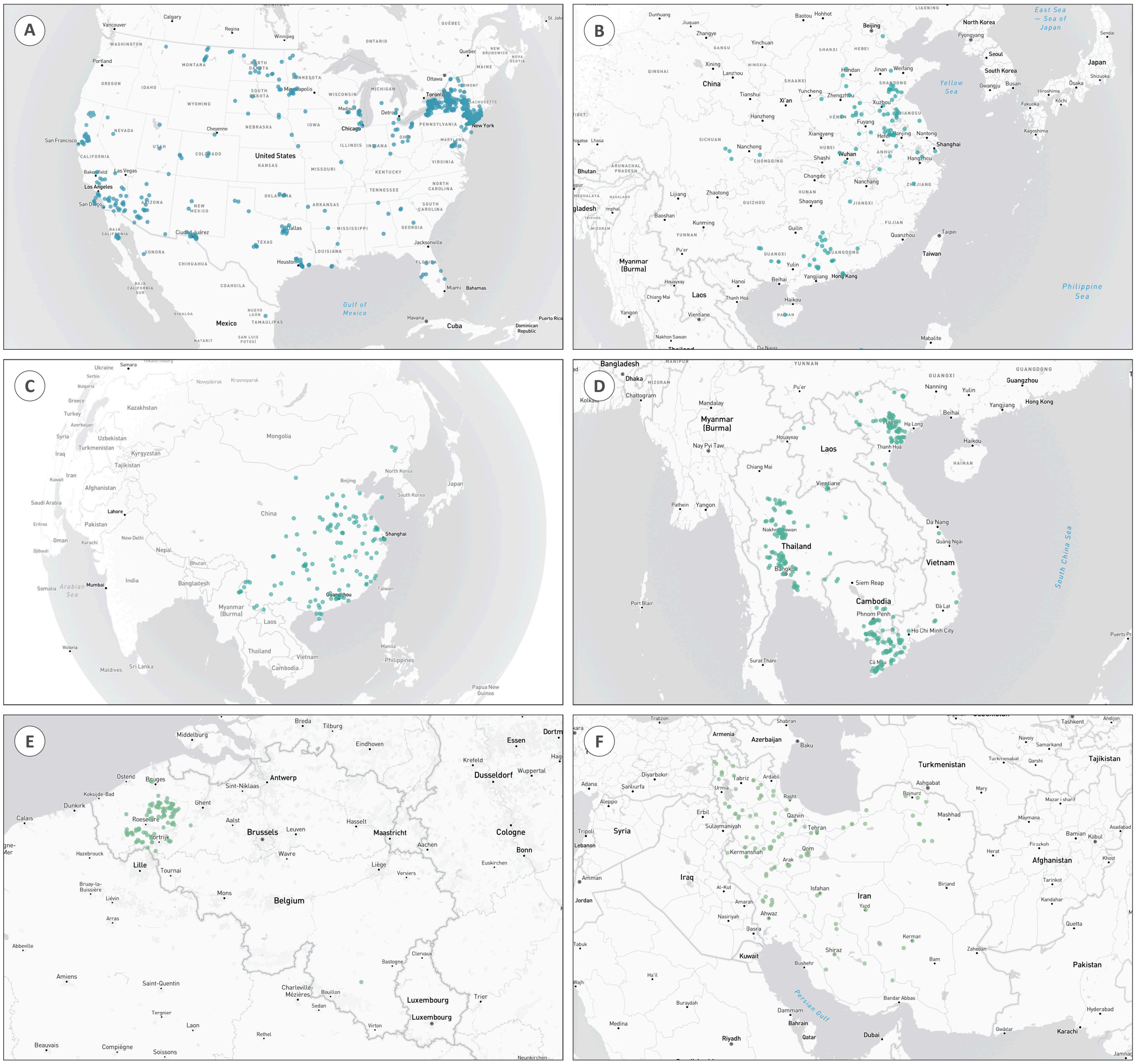

Figure I (part 1/3): sampling maps of the genomic sequences included in the empirical datasets analysed in the present study. (**A**) West Nile virus, North America [1]; (**B**) Porcine deltacoronavirus, China [2]; (**C**) Getah virus, China [3]; (**D**) AIV H5N1, Mekong region [4]; (**E**) AIV H3N1, Belgium [5]; (**F**) Rabies virus (dogs), Iran [6]. Source of the base layer of the maps: Mapbox (www.mapbox.com). The data underlying this figure can be found in https://doi.org/10.5281/zenodo.13984927.

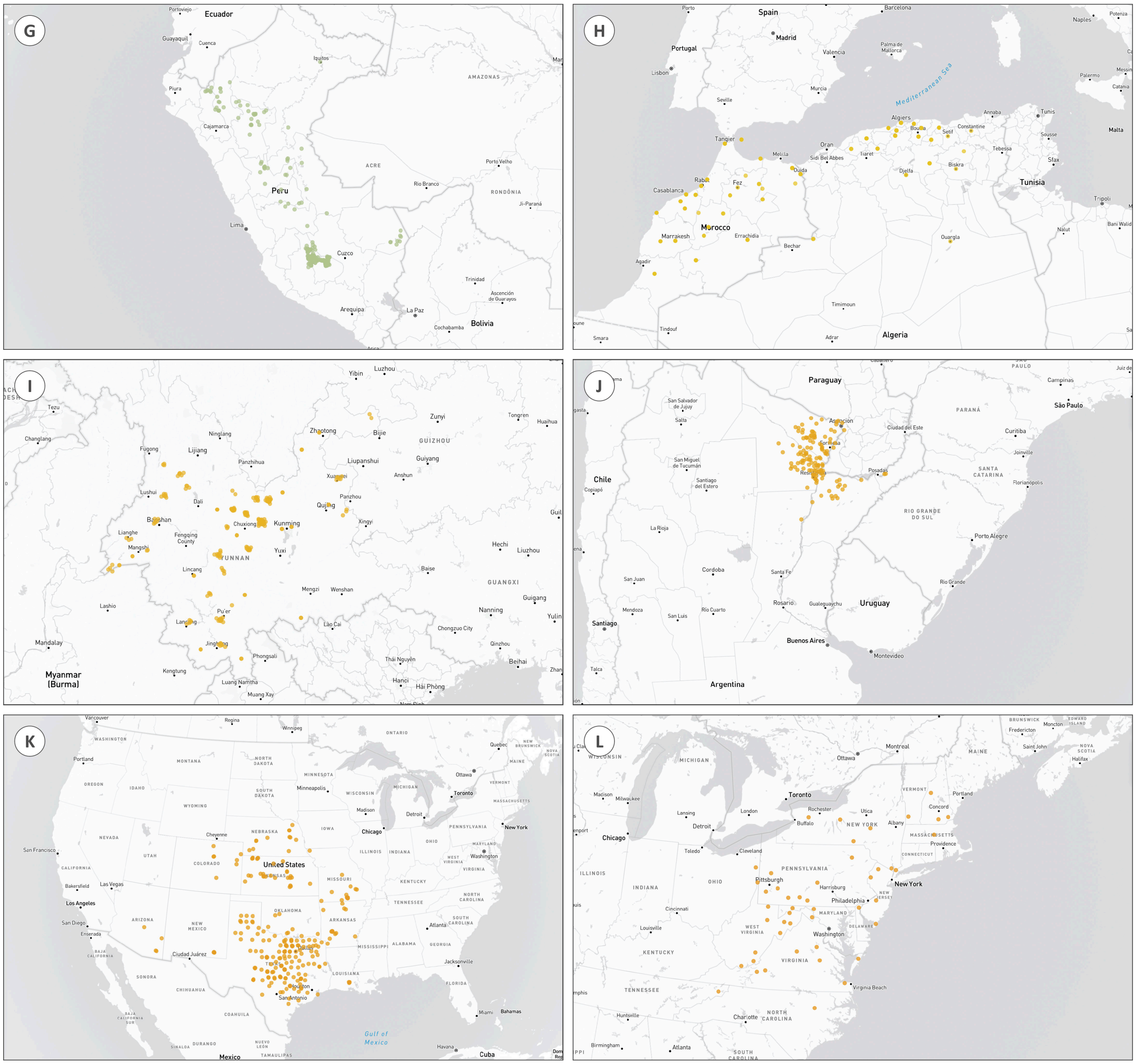

Figure I (part 2/3): sampling maps of the genomic sequences included in the empirical datasets analysed in the present study. (**G**) Rabies virus (bats), Peru [7]; (**H**) Rabies virus (dogs), North Africa [8]; (**I**) Rabies virus (dogs), Yunnan (China) [9]; (**J**) Rabies virus (bats), Argentina [10]; (**K**) Rabies virus (skunks), USA [11]; (**L**) Rabies virus (raccoons), USA [12]. Source of the base layer of the maps: Mapbox (www.mapbox.com). The data underlying this figure can be found in https://doi.org/10.5281/zenodo.13984927.

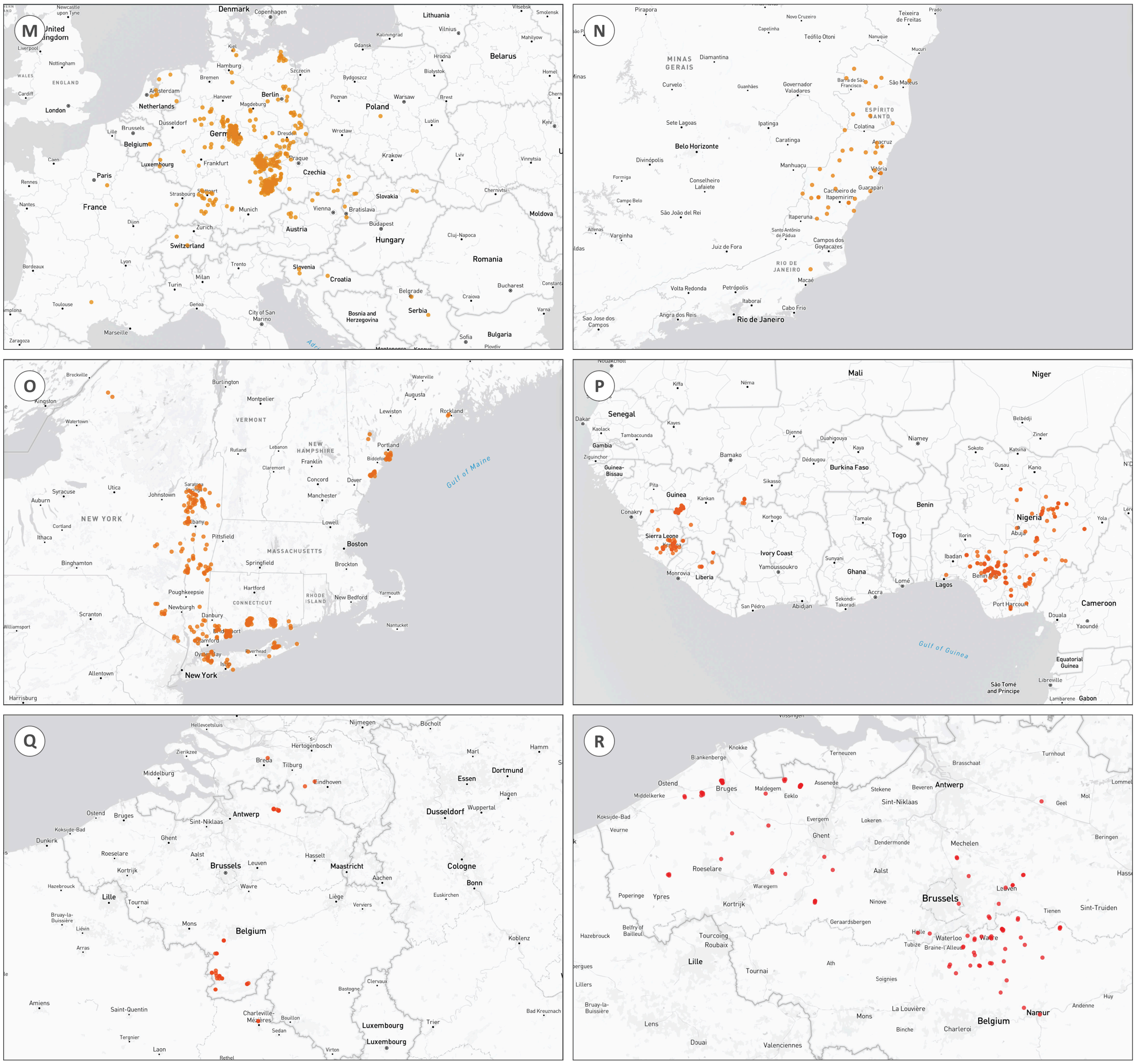

Figure I (part 3/3): sampling maps of the genomic sequences included in the empirical datasets analysed in the present study. (**M**) Tula virus, European clade [13]; (**N**) Rabies virus (bats), eastern Brazil [14]; (**O**) Powassan virus, USA [15]; (**P**) Lassa virus, segment S, Africa [16]; (**Q**) Puumala virus, Belgium [17]; (**R**) Nova virus, Belgium [18]. Source of the base layer of the maps: Mapbox (www.mapbox.com). The data underlying this figure can be found in https://doi.org/10.5281/zenodo.13984927.

Table A: comparison of dispersal metrics estimated for different genomic datasets of viruses spreading in animal populations. The isolation-by-distance (IBD) signal has been estimated by the Pearson correlation coefficient (r_P) between the patristic and log-transformed great-circle geographic distances computed for each pair of virus samples. For each dataset and metric, we report both the posterior median estimate and the 95% highest posterior density (HPD) interval. The extent of the study areas were approximated by the inland area of the minimum convex hull polygon surrounding all the sampling locations of a given dataset. "AIV" refers to avian influenza virus; (*) estimates based on the combined analysis of lineages L1 and L3; (**) estimates based on the analysis of the wild-type strains (see Van Borm *et al*. [19] for further detail); (***) different weighted diffusion coefficient estimates are obtained when solely focusing on phylogeny branches occurring before (113918 km^2/year, 95% HPD = [102250, 136202]) and after (43400 km^2/year, 95% HPD = [38943, 49230]) 2004; which approximately corresponds to the end of what can be characterised as the expansion phase of the West Nile virus outbreak in North America.

Dataset	Weighted diffusion coefficient (km^2/year)	IBD signal (r_P)	Num. of samples	Study area size (km^2)	Reference
Nova virus, Belgium	1 [1, 2]	0.68 [0.64, 0.73]	100	~8,000	Laenen *et al*. (2016) [18]
Puumala virus, Belgium	1 [1, 3]	0.82 [0.75, 0.87]	66	~8,000	Laenen *et al*. (2019) [17]
Lassa virus, segment S, Africa	42 [38, 49]	0.79 [0.76, 0.81]	410	~1,255,000	Klitting *et al*. (2022) [16]
Powassan virus, USA	126 [101, 154]	0.32 [0.24, 0.42]	273	~118,000	Vogels *et al*. (2023) [15]
Rabies virus (bats), eastern Brazil	274 [149, 427]	0.32 [0.20, 0.38]	41	~44,000	Vieira *et al*. (2013) [14]
Tula virus, European clade	399 [282, 506]	0.52 [0.45, 0.55]	380	~1,270,000	Cirkovic *et al*. (2022) [13]
Rabies virus (raccoons), USA	561 [453, 706]	0.09 [0.05, 0.16]	47	~457,000	Biek *et al*. (2007) [12]
Rabies virus (skunks), USA	580 [477, 679]	0.55 [0.46, 0.61]	241	~1,892,000	Kuzmina *et al*. (2013) [11]
Rabies virus (bats), Argentina	721 [550, 909]	0.22 [0.16, 0.29]	131	~142,000	Torres *et al*. (2014) [10]
Rabies virus (dogs), Yunnan (CH)	1064 [75, 1544]	0.06 [0.01, 0.13]	247	~301,000	Tian *et al*. (2018) [9]
Rabies virus (dogs), North Africa	1191 [546, 4844]	0.60 [0.55, 0.63]	250	~667,000	Talbi *et al*. (2010) [8]
Rabies virus (bats)*, Peru	1416 [1086, 1812]	0.74 [0.64, 0.82]	260	~675,000	Streicker *et al*. (2016) [7]
Rabies virus (dogs), Iran	1643 [1307, 2135]	0.30 [0.28, 0.32]	105	~1,052,000	Dellicour *et al*. (2019) [6]
AIV H3N1, Belgium	1794 [1414, 2200]	0.26 [0.14, 0.33]	101	~5,000	Van Borm *et al*. (2023) [5]
AIV H5N1, Mekong region	20647 [16951, 24285]	0.29 [0.18, 0.37]	320	~956,000	Dellicour *et al*. (2020) [4]
Getah virus, China	24930 [17065, 34887]	0.11 [0.06, 0.17]	125	~4,060,000	Zhao *et al*. (2023) [3]
Porcine deltacoronavirus, China	26093 [16753, 36264]	0.21 [0.16, 0.25]	97	~1,967,000	He *et al*. (2020) [2]
Lumpy skin disease virus**	52989 [16749, 99297]	0.75 [0.66, 0.86]	34	~31,658,000	Van Borm *et al*. (2023) [19]
West Nile virus, North America***	58757 [55087, 62105]	-0.02 [-0.04, 0.00]	801	~7,947,000	Dellicour *et al*. (2020) [1]

References

1. Dellicour S, Lequime S, Vrancken B, Gill MS, Bastide P, Gangavarapu K, et al. Epidemiological hypothesis testing using a phylogeographic and phylodynamic framework. Nat Commun. 2020;11: 5620. doi:10.1038/s41467-020-19122-z
2. He W-T, Ji X, He W, Dellicour S, Wang S, Li G, et al. Genomic epidemiology, evolution, and transmission dynamics of porcine deltacoronavirus. Molecular Biology and Evolution. 2020;37: 2641–2654. doi:10.1093/molbev/msaa117
3. Zhao J, Dellicour S, Yan Z, Veit M, Gill MS, He W-T, et al. Early genomic surveillance and phylogeographic analysis of Getah virus, a reemerging arbovirus, in livestock in China. J Virol. 2022;97: e01091-22. doi:10.1128/jvi.01091-22
4. Dellicour S, Lemey P, Artois J, Lam TT, Fusaro A, Monne I, et al. Incorporating heterogeneous sampling probabilities in continuous phylogeographic inference — Application to H5N1 spread in the Mekong region. Bioinformatics. 2020;36: 2098–2104. doi:10.1093/bioinformatics/btz882
5. Van Borm S, Boseret G, Dellicour S, Steensels M, Roupie V, Vandenbussche F, et al. Combined phylogeographic analyses and epidemiologic contact tracing to characterize atypically pathogenic avian influenza (H3N1) epidemic, Belgium, 2019. Emerg Infect Dis. 2023;29: 351. doi:10.3201/eid2902.220765
6. Dellicour S, Troupin C, Jahanbakhsh F, Salama A, Massoudi S, Moghaddam MK, et al. Using phylogeographic approaches to analyse the dispersal history, velocity, and direction of viral lineages – application to rabies virus spread in Iran. Mol Ecol. 2019;28: 4335–4350. doi:10.1111/mec.15222
7. Streicker DG, Winternitzc JC, Satterfield DA, Condori-Condori RE, Broos A, Tello C, et al. Host-pathogen evolutionary signatures reveal dynamics and future invasions of vampire bat rabies. Proceedings of the National Academy of Sciences of the United States of America. 2016;113: 10926–10931. doi:10.1073/pnas.1606587113
8. Talbi C, Lemey P, Suchard MA, Abdelatif E, Elharrak M, Jalal N, et al. Phylodynamics and human-mediated dispersal of a zoonotic virus. PLoS Path. 2010;6: e1001166.
9. Tian H, Feng Y, Vrancken B, Cazelles B, Tan H, Gill MS, et al. Transmission dynamics of re-emerging rabies in domestic dogs of rural China. PLoS Pathogens. 2018;14: e1007392. doi:10.1371/journal.ppat.1007392
10. Torres C, Lema C, Gury Dohmen F, Beltran F, Novaro L, Russo S, et al. Phylodynamics of vampire bat-transmitted rabies in Argentina. Molecular Ecology. 2014;23: 2340–2352. doi:10.1111/mec.12728
11. Kuzmina NA, Lemey P, Kuzmin IV, Mayes BC, Ellison JA, Orciari LA, et al. The phylogeography and spatiotemporal spread of south-central skunk rabies virus. PLoS One. 2013;8: e82348.
12. Biek R, Henderson JC, Waller LA, Rupprecht CE, Real LA. A high-resolution genetic signature of demographic and spatial expansion in epizootic rabies virus. Proceedings of the National Academy of Sciences of the United States of America. 2007;104: 7993–7998.
13. Cirkovic V, Dellicour S, Stamenkovic G, Siljic M, Gligic A, Stanojevic M. Phylogeographic analysis of Tula hantavirus highlights a single introduction to central Europe. Virus Evolution. 2022;8: veac112. doi:10.1093/ve/veac112

14. Vieira LFP, Pereira SRFG, Carnieli Jr P, Tavares LCB, Kotait I. Phylogeography of rabies virus isolated from herbivores and bats in the Espírito Santo State, Brazil. Virus Genes. 2013;46: 330–336. doi:10.1007/s11262-012-0866-y
15. Vogels CBF, Brackney DE, Dupuis AP, Robich RM, Fauver JR, Brito AF, et al. Phylogeographic reconstruction of the emergence and spread of Powassan virus in the northeastern United States. Proc Natl Acad Sci USA. 2023;120: e2218012120. doi:10.1073/pnas.2218012120
16. Klitting R, Kafetzopoulou LE, Thiery W, Dudas G, Gryseels S, Kotamarthi A, et al. Predicting the evolution of the Lassa virus endemic area and population at risk over the next decades. Nat Commun. 2022;13: 5596. doi:10.1038/s41467-022-33112-3
17. Laenen L, Vergote V, Vanmechelen B, Tersago K, Baele G, Lemey P, et al. Identifying the patterns and drivers of Puumala hantavirus enzootic dynamics using reservoir sampling. Virus Evolution. 2019;5: vez009. doi:10.1093/ve/vez009
18. Laenen L, Dellicour S, Vergote V, Nauwelaers I, De Coster S, Verbeeck I, et al. Spatio-temporal analysis of Nova virus, a divergent hantavirus circulating in the European mole in Belgium. Molecular Ecology. 2016;25: 5994–6008. doi:10.1111/mec.13887
19. Van Borm S, Dellicour S, Martin DP, Lemey P, Agianniotaki EI, Chondrokouki ED, et al. Complete genome reconstruction of the global and European regional dispersal history of the lumpy skin disease virus. J Virol. 2023;97: e01394-23. doi:10.1128/jvi.01394-23
