# Supplementary material for: How fast are viruses spreading in the wild?
Source: PLoS Biol. 2024 Dec 3;22(12):e3002914. doi: 10.1371/journal.pbio.3002914 (PMC11614233; doi:10.1371/journal.pbio.3002914)
Supplement: Attachment — Submitted filename: Response_to_Reviewers.pdf [file pbio.3002914.s002.pdf]

Reviewer 1

This is a straightforward paper that looks at several metrics of dispersal computed from phylogeographic trees, and applies the metrics to 19 viral datasets from the literature. So, the first part of the paper evaluates these metrics on simple branching random walk simulations, and shows that one of them - the "weighted lineage dispersal velocity" (WLDV) - is sensitive to the sample size, and hence not a consistent estimator of anything. (The underlying reason for this is explained with some math.) The second part of the paper evaluates the remaining two metrics on the viral datasets, and finds a surprising and interesting array of results.

I am of two minds about this paper. I really like the premise of the paper: evaluation of these metrics of dispersal seems important, and application to a wide variety of viral datasets is very interesting, and the diversity of results found on those datasets is intriguing. In particular, WLDV is used at least occasionally in the literature, so it seems important to point out its shortcomings. However, a major shortcoming of the paper is that it does not explore the effects of sampling *location* (only sample size), which leads me to question both main conclusions (i.e., that the other two metrics are robust, and that the comparison between datasets is meaningful). To be clear, I think this is a useful and interesting paper, but given this substantial gap between the results and the real world on both fronts, doesn't feel like the sort of results I'd expect in PLoS Biology. That said, it may be that I'm missing the big picture; if so, then I recommend the authors emphasize the importance and/or significance of the results.

In more detail: all simulation results in the paper assume that sampling occurs independently of location. This is sufficient to demonstrate the point that WLDV is sensitive to number of samples, but does not demonstrate that the other two metrics are robust to this aspect of sampling. In fact, I strongly suspect that the other metrics (at least, WDC) are strongly affected by spatial sampling. (Consider the extreme case where all samples came from nearly the same location; then ancestral locations would also be inferred to be from that same location as well, and so the inferred diffusivity would be very small.) To be fair, the authors do not misrepresent their findings (e.g., the abstract says ``robust to the number of samples''), and there is a paragraph in the Discussion about this limitation. So, I think these results are useful and not wrong, just lacking in depth.

The empirical results are also much harder to interpret given this caveat: since all data is from other publications, there is no description of sampling, so one cannot even guess whether spatial sampling might affect results. (Ideally, there would be maps of sampling locations for each outbreak.) It may well be that the results presented are not strongly affected by sampling, but again, these are lacking in depth and thus don't make the impact they might have otherwise.

<u>Answer</u>: Thank you for raising an important point. We agree that the impact of spatial sampling bias on continuous phylogeographic inference is important to study, but we considered recent work to have largely addressed this (Kalkauskas *et al*. 2021, PMID: 33406072; Guindon & De Maio 2021, PMID: 34930835). However, we realise that the studies have not specifically focused on the metrics we investigate here. Therefore, to address the Reviewer's comment, we have now analysed and integrated a third set of simulations performed by simulating a RRW diffusion process along the branches of an empirical phylogenetic tree (in our case the maximum clade credibility tree obtained from the continuous phylogeographic reconstruction of the WNV spread in North America based on >800 near full genome sequences; PMID: 33159066). As described in the revised version of our manuscript, this additional simulation framework enables us to take phylogenetic uncertainty into account (see below), and also to explore scenarios of sampling bias. In the two original simulation approaches (respectively based on a

birth-death and a coalescent simulation process), the different datasets are directly obtained by subsampling the simulated tree topology, which does not readily lend itself to studying the impact of a sampling bias. In the third simulation procedure, we now re-perform the actual continuous phylogeographic inference based on the coordinates simulated at the tip nodes, which in turn allows exploring the effect of sampling bias on the outcome of such an inference. The implementation of scenarios of sampling bias has now been described as follows in the Material & Methods section:

"*Finally, we also exploited the RRW simulations conducted along a tree topology to explore the impact of two scenarios of sampling bias on the robustness of the three dispersal metrics considered in our study. For this purpose, we used the simulation procedure described above, but instead of randomly subsampling each simulation to obtain datasets associated with various sampling intensity, we performed a subsampling corresponding to a scenario of spatial sampling bias where only a restricted area of the spatial distribution of the virus spread was sampled. In the case of the spatially unconstrained simulations, the subsampling was performed to only retain all tip nodes simulated east of the tree root location. In the case of the spatially constrained simulations, the subsampling was performed to only retain all tip nodes simulated further east than the 86th West meridian, which roughly corresponds to a subsampling restricted to the first eastern third of the contiguous USA area that was invaded by the West Nile virus.*"

We further elaborate on these results, which do not indicate a pronounced sampling bias, in reply to the next comment, but we acknowledge that we only examine limited scenarios of sampling bias.

Other points:

In the simulations, there is no phylogenetic inference step. This is because "adding a genomic sequence simulation step would have also drastically increased the computation time and resources required to conduct the analyses". (I assume this means that the Bayesian inference of phylogeny would be resource-intensive, since simulating even large genomes on a tree is trivial.) This may be a fair point, but if this paper is to be a comprehensive assessment of the other two metrics, rather than just a takedown of WLDV, then phylogenetic error should also be assessed (at least in a few simulations, or perhaps in a non-Bayesian framework).

Answer: The Reviewer is correct that the tree inference would be the resource-intensive task. To address the Reviewer's concern, we have now designed a simulation and inference procedure that accounts for empirical phylogenetic uncertainty while avoiding the computational burden of sequence-based inference. Specifically, we conducted a third set of simulations by simulating RRW diffusion processes along the branches of a single empirical phylogenetic tree and then integrated over an empirical set of trees in the inference. This is described as follows in the Materials and Methods section of the revised manuscript:

"*Thirdly, we also conducted RRW simulations along the branches of an empirical phylogenetic tree: the maximum clade credibility (MCC) tree obtained from the continuous phylogeographic reconstruction based on 801 West Nile virus genomes collected between 1999 and 2016 in North America [31] (Fig. S7). To this end, we used the "simulatorRRW1" function implemented in the R package "seraphim" to conduct RRW simulations along time-scaled tree topologies [19]. Specifically, we simulated distinct forward-in-time RRW diffusion processes along the branches of the empirical MCC tree using the precision matrix parameters and root node location inferred by the original continuous phylogeographic analysis, this location having been inferred in the vicinity of New York City [19]. These simulations were either spatially unconstrained or constrained to the contiguous area of the United States, and five distinct simulations*

were conducted for each of these two categories of simulations (see Figure S7 for the example of one spatially constrained simulation). Again, we subsequently subsampled each simulation to obtain time-scaled phylogenies only including 500, 450, 400, 350, 300, 250, 200, 150, 100, and 50 tip nodes associated with sampling coordinates simulated by the RRW diffusion process along the original MCC tree. In order to investigate the robustness of the dispersal metrics to the sampling effort while considering the uncertainty associated with the phylogenetic inference, we then performed a new continuous phylogeographic reconstruction based on each resulting dataset. To incorporate the uncertainty associated with phylogenetic inference, we performed all phylogeographic reconstructions while integrating over 100 posterior trees retrieved from the phylogeographic inference previously conducted on the actual West Nile virus dataset [31]. In this way, we account for tree topology uncertainty when assessing the robustness of the dispersal metrics to the sampling effort without the need to confront inference from sequence data."

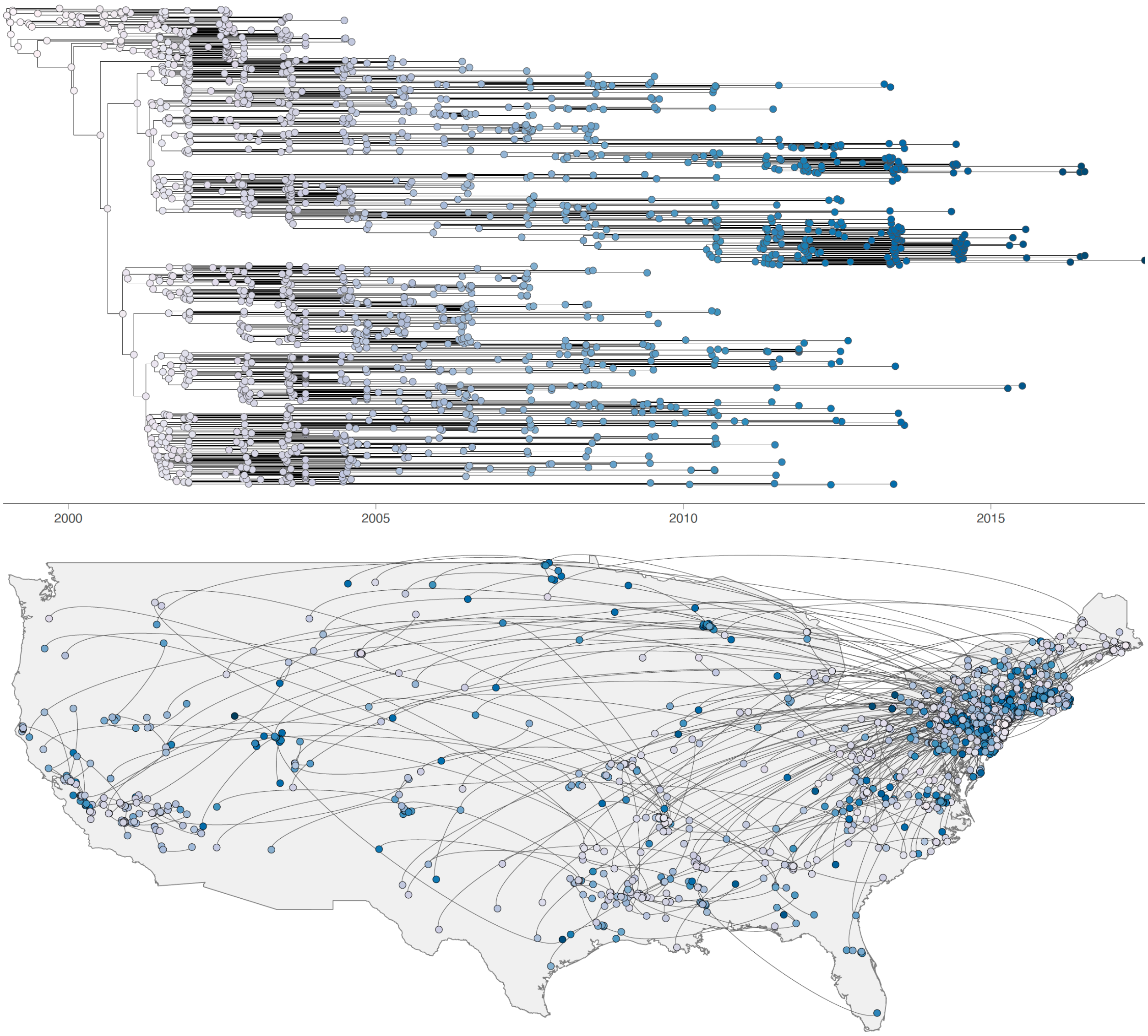

***Figure S7: example of a continuous phylogeographic simulation based on a relaxed random walk (RRW) diffusion process simulated along the branches of the maximum clade credibility (MCC) tree obtained from the continuous phylogeographic reconstruction itself based on West Nile virus genomes collected between 1999 and 2016 in North America [30].** Both graphs display the phylogenetic tree resulting from a single simulation, with its time-scaled tree visualisation in the top panel and its*

mapped visualisation in the bottom panel. Tree nodes are coloured according to time, with internal and tip nodes coloured according to their time of occurrence and collection time, respectively.

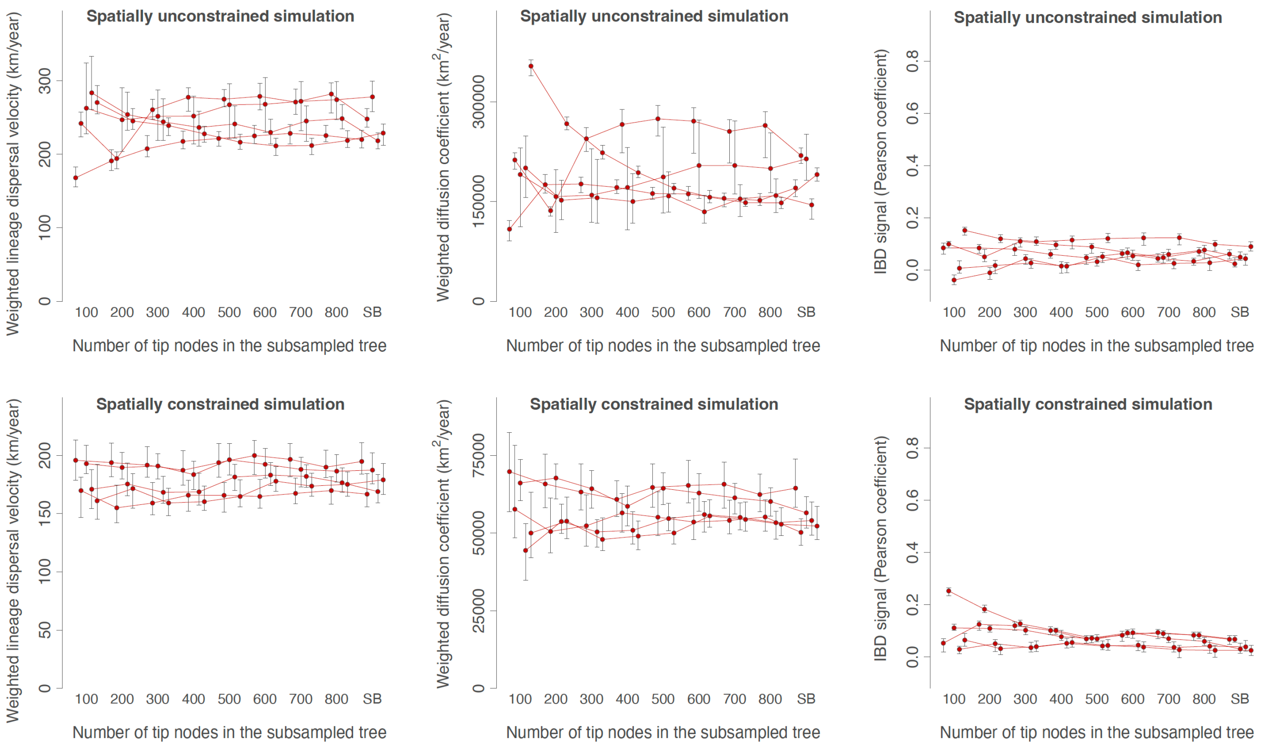

Figure S8: robustness of lineage dispersal metrics to the sampling effort and bias while incorporating the uncertainty associated with the phylogenetic inference. *We report the three dispersal statistics under investigation — the weighted lineage dispersal velocity (WLDV), the weighted diffusion coefficient (WDC), and the isolation-by-distance signal (r_P) metrics — estimated from a set of posterior trees each time sampled from a continuous phylogeographic inference based on datasets simulated for the West Nile virus spread in North America [31] (see the text for further detail on the simulation process illustrated in Figure S7). In the different graphs, the vertical intervals correspond to the 95% highest posterior density (HPD) interval of dispersal metrics estimated on 100 posterior trees, and the red dots to the corresponding median estimates. Simulations were either spatially unconstrained (first row of graphs) or constrained (second row of graphs) to the mainland United States (excluding Alaska). For each of these two categories of simulations, we first considered datasets resulting from various sampling intensities, ranging from 100 to 800 sampled genomes randomly sampled within the original West Nile virus dataset. Secondly, for each category of simulations, we also considered a scenario of sampling bias (SB), which corresponded to a sampling restricted to all genomes sampled east of the tree root location for the unconstrained simulations (corresponding, on average, to half of the simulated samples), and all genomes further east of the 86th West meridian for the spatially constrained simulations. In the latter case, with a root location inferred in the New York city area for the West Nile virus invasion in North America, it roughly corresponds to a subsampling restricted to the first eastern third of the invaded area across mainland USA. In the different panels, the red line segments link the results obtained for a distinct initial RRW simulation conducted along the MCC tree.*

We now report the new results as follows in the revised manuscript:

"In addition to the simulations conducted with a birth-death or coalescent approach, we have also conducted continuous phylogeographic simulations by simulating RRW diffusion processes along the branches of an empirical phylogenetic tree, i.e. the maximum clade credibility (MCC) tree previously obtained from the phylogeographic analysis of the West Nile virus in North America [31] (Fig. S7; see also the Materials and Methods section for further detail). The specific aim of these additional simulations is to assess the robustness of the dispersal metrics to the sampling effort, as well as to a scenario of sampling bias, but this time also considering the uncertainty associated with phylogenetic inference. As illustrated in Figure S8, our results indicate that when explicitly considering the uncertainty associated with the inference of the time-scaled tree topologies, the impact of various scenarios of sampling intensity on the dispersal metrics is less evident. Indeed, the posterior distribution of the dispersal metric estimates appears

to be often quite large, sometimes preventing a clear distinction of any impact of sampling intensity on these estimates. Furthermore, in order to take into account the uncertainty related to phylogenetic inference, this additional simulation framework required to re-perform a continuous phylogeographic inference based on each subset of geo-referenced tips sampled from the original simulation for generating various scenarios of sampling intensity (see the Materials and Methods section for further detail). Because of this additional inference step, it seems that the random selection of sequences has often more impact on the dispersal metric estimates than the number of samples that were subsampled, especially when the sampling size decreases (Fig. S8). The later result underlines the random impacts of the sampling itself, independently of the impact of the sampling size. As introduced above, we have also used the same simulation framework to estimate the dispersal metrics from simulations based on a scenario of sampling bias where only a restricted area of the spread has been sampled. Similarly, our results based on this simulation scenario and explicitly considering phylogenetic uncertainty do not indicate a clear impact of sampling bias on the estimates (Fig. S8)."

The mathematical explanation of why WLDV depends on sample size (around l.147-158) is nice and simple but very awkwardly explained IMO: I think the explanation could be simplified and smoothed out substantially. Also, it might be good to relate this to the literature: a name for the observation on l.157 is that the "total variation of a Brownian path is infinite", and a name for the observation on l.162 is that "Brownian motion is an infinitely divisible process with constant quadratic variation". (However, these points aren't going to help non-mathematical readers understand the argument.) Also note that the equation on l.162 applies to any random walk with independent increments of finite variance, not just Brownian, since the variance of the sum of independent things is the sum of their variances.

Answer: Thank you for the suggestions and the pointers to the literature. We have now modified this paragraph, adding general references to the linear and quadratic variation behaviours of the Brownian motion as suggested. We keep the special case of the univariate process sampled linearly in time for illustration purposes, as in this case the computations are more straightforward to write down and explain. The updated paragraph now reads as follows:

"*From a mechanistic perspective, the lack of robustness of the lineage dispersal velocity metric can actually be expected from a Brownian Motion process, which has paths with infinite variation on any interval (see e.g., Corollary 2.17 in [30]). This implies that, the more densely sampled the trajectory is, the larger the WLDV becomes, making the estimation of an average speed sampling inconsistent. In contrast, the Brownian motion has finite quadratic variation (see e.g., Proposition 2.16 in [30]), so that the WDC, that uses quadratic distances instead of linear ones, should not be sensitive to the number of samples. To illustrate these theoretical expectations, we consider the case of a strict one-dimensional Brownian diffusion process of position X along a time interval equal to 1, with N being the total number of regular time intervals dt = 1/N, d_i the distance travelled during time interval i, X_i the position at the end of interval i, and $X_{pa(i)}$ the position at the end of the previous time interval. [...]*"

The authors may want to cite Neigel and Avise, Genetics, 1993, who have the same goal with essentially the same type of data.

Answer: Thank you for the suggestion, we now also cite their work.

Finally a note: the scheme of randomly rotating two independent Cauchys seems fine to me; however for future reference a less ad hoc method of obtaining a “bivariate Cauchy” is in my opinion to use the “scale mixture of Normals” approach, i.e., to divide a standard bivariate Normal by the square root of a Gamma with the appropriate parameters (search “Cauchy scale mixture of Normals”).

Answer: Well noted, thank you for the suggestion. This approach is indeed similar to the parameterisation used for the bivariate RRW process in BEAST. We chose this rotation approach to mirror the simulation framework in the Brownian motion case.

Minor comments:

l.21: “allowing to unveil”: “unveil” is imprecise; also, the grammar is wrong (“allowing us to unveil” or “which can unveil” would be better)

Answer: Thank you for your comment, we have now proposed an alternative version of that sentence: “*These spatially explicit reconstructions can subsequently be used to estimate dispersal metrics that can inform on the dispersal dynamics and the capacity to spread among hosts*”.

l.22: “accuracy of dispersal insights” -> “accuracy of dispersal estimates”

Answer: Modification has been performed.

l.24: “implement a simulation framework” -> “use simulations”

Answer: Modification has been performed.

l.52: “allows estimating” -> “allows estimation of”

Answer: Modification has been performed.

l.69: “connected *by* the air traffic network”, maybe?

Answer: Yes, thank you for the suggestion; modification has been performed.

l.73: “by essence” -> “in essence”

Answer: Modification has been performed.

l.87: “on each phylogeny branch” -> “on the i-th branch of the phylogeny”

Answer: Modification has been performed.

l.101-102: “longitudinal and latitudinal displacements are randomly drawn from a Cauchy distribution” - this is not accurate, because of the (important) random rotation later mentioned. Please rephrase.

Answer: Thank you for your comment, we have now completed this sentence as follows: “*longitudinal and latitudinal displacements are randomly drawn from a Cauchy distribution before randomly rotating the resulting displacement around its origin*”.

l.103: “The example” -> “An example”, maybe?

Answer: Modification has been performed.

l.119: “We have then estimated the three dispersal metrics” - as defined, your metrics are quantities computed from data, and so are “computed”, not “estimated”. (They are statistics, not some underlying parameter you are trying to estimate.)

Answer: Modification has been performed.

l.248: “relatively important diffusion coefficient” -> do you mean “large”?

Answer: Yes indeed, modification has been performed.

l.275: “adding a genomic sequence simulation step would have also drastically increased the computation time and resources required to conduct the analyses” - perhaps clarify that the step that would take substantial time is that of Bayesian inference; generation of genome sequences is trivial (e.g., with msprime).

Answer: Good point, we have now modified that sentence as follows: “*From a computational perspective, adding a genomic sequence simulation step would also have drastically increased the computation time and resources required to conduct each Bayesian phylogeographic analysis.*”

l.295: “the diffusion coefficient allows estimating a diffusion coefficient as the invaded area per unit of time” - awkward; also, although the expression involves distance-squared, I don't think the best interpretation of this is as “area”.

Answer: Thank you for your comment, this sentence has now been rephrased as follows: “*while the diffusion coefficient allows estimating dispersal capacity, the IBD signal metric will quantify to what extent the geo-referenced phylogenies are spatially structured, i.e. the tendency for phylogenetically-related lineages to evolve in a close vicinity.*”

l.301: “between *infection locations of* two successive hosts”

Answer: Modification has been performed.

l.308: “The comparative framework initiated in the present study” - the comparisons are excellent; the framework is not new.

Answer: We fully agree, and this is indeed not what we intended to say. We rewrote that sentence to avoid implying a novelty on the framework, but just that it can be completed in further studies: “*Future studies applying continuous phylogeographic analyses to new genomic datasets can further build on the comparison of dispersal metric estimates that we initiated here.*”

l.320: “Unfortunately, fully Bayesian phylogeographic approaches” - why is it important for these analyses that the inference be fully Bayesian?

Answer: We acknowledge that this part of the text was not developed enough. The emphasis should actually not be on the “full Bayesian” aspect but on the interest of explicitly taking into account the phylogenetic uncertainty when conducting a phylogeographic reconstruction. In the context of Bayesian phylogeographic inference, this can be achieve in two different ways: by performing an integrated Bayesian inference where the phylogenetic and phylogeographic history are jointly performed, which does correspond to a common practice in the field; or alternatively by first performing a Bayesian

phylogenetic inference and subsequently conducting phylogeographic reconstructions along several trees sampled from the first posterior distribution. This aspect is now clarified in the text.

l.337: delete “Of note, we defined a sampling window so that”

Answer: We would prefer to maintain this detail within our Materials and Methods section to be totally transparent regarding the implemented simulation framework.

l.338: “In between the two,” - between the two what?

Answer: This was indeed not sufficiently clear, we have now re-phrased it as follows: “*in between the probability to give birth and the probability to be sampled*”.

l.341: “in a normal distribution of” -> “from a Normal distribution with”

Answer: The modification has been done.

l.340 and l.342: the phrase “stochastically defined” is odd: better “sampled”, maybe?

Answer: Thank you for the suggestion, modifications have been performed accordingly.

l.340: The description here is not correct, or at least confusing, since it first says that d_x and d_y are the horizontal and vertical displacements, but then it says that these are randomly rotated (and so hence aren't actually the horizontal and vertical displacements). It might be better to (a) omit the random rotation for the Normal (even if you did the random rotation in your code, since these are mathematically equivalent); and (b) for the Cauchy say you picked a random angle and then did two independent, orthogonal displacements in the directions defined by that angle.

Answer: Thank you for pointing out this aspect. We have now re-written this part of the text to explicitly describe a two-steps procedure (first drawing d_x and d_y from a distribution, and then rotating the resulting displacement around its origin).

l.343: “in a Cauchy distribution” -> “from a Cauchy distribution”

Answer: Modification has been performed.

l.343: Unclear what the purpose of the citation is - explain.

Answer: This citation has now been discarded to avoid any confusion.

l.347: “gradient” -> “direction”

Answer: Modification has been performed.

Reviewer 2

The authors' efforts to directly address the impact of sampling heterogeneity is appreciated and is highly relevant to a major challenge to epidemiologic studies incorporating genomic sequencing data. However, the efforts to examine the robustness of various methods have been applied to animal data. This is understandable from a methodological standpoint - animal to animal transmission is more likely to be constrained by immediate geographic proximity, unlike humans who travel by air and other modes. However, the problem at hand (whether sampling depth impacts inference for these methods) seems to be a more relevant problem to human studies, particularly outbreak investigations where many connected cases are sampled and sequenced. Any further connection that can be made to applications in human data of this first component would strengthen the paper.

Answer: While indeed frequently used to reconstruct the spread of viruses in animal populations, continuous phylogeographic analyses have also been performed to reconstruct the dispersal history of viruses spreading in human populations (see e.g. PMID: 29884821, PMID: 31809523, PMID: 35760080), especially when the dispersal process maintains a sufficient dependence on geographic distance (e.g. over limited spatial scales). Overall, the investigations conducted in our study are therefore also relevant when applying a relaxed random walk diffusion model to viruses transmitting among humans. This aspect is now explicitly stated in the text: "*At relatively large scales, dispersal dynamics of viruses primarily spreading within human populations are generally better captured by discrete diffusion processes involving the rapid exchange of viral lineages among remote locations — a typical pattern for remote locations mainly connected by the air traffic network (see e.g. [16,17]). This is for instance the case for seasonal human influenza virus H3N2, for which dispersal at the global scale has been demonstrated to be better predicted by international air travel than geographic distances [18]. While continuous phylogeographic analyses can also be performed to reconstruct the dispersal history of viruses spreading in human populations (see e.g. [19–21]), our study focuses on viruses circulating in animal populations to target dispersal processes that are relatively strongly dependent on geographic distance. Such dispersal patterns can in essence be modelled through a continuous diffusion process, such as the relaxed random walk (RRW) diffusion model implemented in the software package BEAST 1.10 [22], which is often used for continuous phylogeographic inference.*"

Secondly, the evaluations of geographic spread are interesting and valuable. These simulations, and the methods presented, are a helpful basis for future studies of phylogeography of viruses. These are particularly valuable for future studies of animal viruses that are emerging, for which methods to quickly detect changes in spread dynamics are needed.

Answer: Thank you very much for the positive feedback on our work.
