# Supplementary material for: How fast are viruses spreading in the wild?
Source: PLoS Biol. 2024 Dec 3;22(12):e3002914. doi: 10.1371/journal.pbio.3002914 (PMC11614233; doi:10.1371/journal.pbio.3002914)
Supplement: Attachment — Submitted filename: Response_to_Reviewers.pdf [file pbio.3002914.s003.pdf]

Reviewer 1

Thanks to the authors for the additional work put into this manuscript, which I think has improved it. However, this additional work hasn't really changed my initial opinion of the paper: I think it is a useful and interesting paper, but some more depth and careful analysis would be necessary (in my view) to draw strong conclusions from the results. I think this could be fixed with relatively little additional background provided by the authors.

In more detail: the paper still does not meaningfully investigate the impact of spatially heterogeneous sampling: there is a single plot that examines a single example of spatially restricted sampling that's probably the least worrisome possible situation. The paper has made the issue more obvious, including with citations to other papers discussing the point, and I don't think anyone will misconstrue this paper as concluding that the metrics are robust to changes in spatial sampling. However, this is still a concern because the main conclusion of the paper (see the title and abstract) has to do with the interpretation of empirical datasets, for which there is absolutely no assessment or communication of spatial sampling. I suspect that the authors have some good reason to think that this is unlikely to substantially affect the results (based on experience, perhaps?).

Answer: We do not have such prior experience unfortunately, and fully agree with the Reviewer that the heterogeneous sampling effort inherent to all genomic datasets will always impact any outcome of a phylogeographic reconstruction (as well documented in several previous studies, including specific investigations of continuous phylogeographic inference; PMID: 33406072, PMID: 34930835). The primary objective of our study is not to re-assess the impact of *spatial sampling bias* on the outcomes of phylogeographic inference but to point out that, when only dealing with *heterogeneous sampling intensities* (i.e. various sampling sizes), some dispersal metrics that can be estimated from these phylogeographic reconstructions are already not robust. Yet, we totally acknowledge that, considering its impact on any phylogeographic inference, spatial sampling bias — which, this being said, is not easy to assess given that the actual spatial distribution of individuals/infectious cases is frequently unknown — will also have an impact on any dispersal metric computed from a phylogeographic reconstruction. To further underline this aspect, we have now made this point explicit in the Abstract (see below) and have now also expanded the relevant part of the Discussion with a more in-depth treatment of previous investigations of the impact of spatial sampling bias and their implications for the dispersal capacity metric (WDC) considered in our study (see also our answer below).

Modified abstract: "*Genomic data collected from viral outbreaks can be exploited to reconstruct the dispersal history of viral lineages in a two-dimensional space using continuous phylogeographic inference. These spatially explicit reconstructions can subsequently be used to estimate dispersal metrics that can be informative of the dispersal dynamics and the capacity to spread among hosts. Heterogeneous sampling efforts of genomic sequences can however impact the accuracy of phylogeographic dispersal metrics. While the impact of spatial sampling bias on the outcomes of continuous phylogeographic inference has previously been explored, the impact of sampling intensity (i.e., sampling size) when aiming to characterise dispersal patterns through continuous phylogeographic reconstructions has not yet been thoroughly evaluated. In our study, we use simulations to evaluate the robustness of three dispersal metrics — a lineage dispersal velocity, a diffusion coefficient, and an isolation-by-distance signal metric — to the sampling intensity. Our results reveal that...*"

A solution that would have made me satisfied would be providing maps of sampling locations in the supplement and some discussion of how sampling scheme might affect results. I am not suggesting the authors perform an exhaustive evaluation of how various sampling schemes affect these metrics, as that would be a whole new paper.
To be clear: spatial sampling *definitely does* affect all metrics computed here, and many empirical datasets are strongly spatially biased -- for instance, all samples from two large cities, or along one highway. Roughly speaking, positive spatial autocorrelation of sampling probability should reduce the estimated dispersal distance -- if, for instance, most samples came from a few spatially restricted areas -- and negative spatial autocorrelation should increase it -- if, for instance, available samples were thinned to avoid sequencing lots of samples from the same location. The situation simulated in the current paper almost entirely avoids either of these issues, as uniform sampling in a large sub-range of the region shouldn't affect things much at all. Again, perhaps this is not a problem for the empirical datasets used, but in a paper whose topic is bias induced by sampling I would have expected something more than a sentence in the Discussion.

Answer: While spatial sampling bias is indeed not the scope of our study, we understand the concern of the Reviewer. In addition to previous modifications of our study related to this (including the addition of sampling bias scenarios in our new simulation framework taking phylogenetic uncertainty into account), we have now further integrated discussion elements on the potential impact of spatial sampling bias when characterising dispersal dynamics of lineages through continuous phylogeographic inference. First, and as already mentioned above, we have now also included a more in-depth discussion of the implications of previous investigations of the impact of spatial sampling bias (PMID: 33406072, PMID: 34930835) on the dispersal capacity metric (WDC) considered in our study:

"As introduced above, we have also used these simulations of RRW diffusion processes along empirical tree branches to estimate the dispersal metrics from simulations based on a scenario of sampling bias where only a restricted area of the spread has been sampled (see the Materials and Methods section for further detail). Such a sampling bias scenario has previously been shown to strongly affect continuous phylogeographic reconstructions [33]. Again, our results based on this simulation scenario and explicitly considering phylogenetic uncertainty do not indicate a clear impact of sampling bias on the estimates (Fig. S8). So with this simulation framework, we do not manage to distinguish a clear difference between the dispersal metrics estimated for the scenarios of various sampling intensity and sampling bias. The impact of such spatial sampling bias on the outcomes of continuous phylogeographic inference has been more thoroughly investigated in previous studies [8,33]. For instance, Kalkaukas and colleagues have explored the impact of three sampling bias scenarios on the estimation of the squared net lineage displacement per unit of time (σ^2, referred to as the "diffusion parameter" in their work [33]), which is directly related to a metric like WDC investigated here (see above). Their analyses reveal that all considered scenarios of sampling bias — a central sampling bias, a diagonal sampling bias, and a one-side sampling bias scenario similar to the one explored in this study — lead to an underestimation of the diffusion parameter. Further confirmed by additional investigations of sampling bias scenarios by Guindon & De Maio [8], these results thus indicate that sampling bias can lead to an underestimation of the dispersal capacity as quantified by a metric like WDC."

Second, and as suggested by the Reviewer, we now provide a sampling map for each empirical dataset (Figure S9, in three parts; see part 1/3 below). The heterogeneous spatial distribution of collected samples included in those datasets is now explicitly treated in our Discussion section:

"Finally, while we have previously established that the two dispersal metrics estimated on those empirical datasets appear to be robust to the sampling intensity, they remain impacted by sampling bias like any outcome of a phylogeographic inference. As reported in Figure S9, we see that the sampling maps of the different empirical datasets display an important variability in terms of sampling patterns, with a varying degree of sampling site clustering. The associated sampling bias is however difficult to evaluate in practice given that the actual spatial distribution of individuals/infectious cases is frequently unknown, especially when studying pathogens spreading in wild animal populations. We can nevertheless clearly speculate that infected areas were likely under or even unsampled for a good number of those datasets. As established in previous works [8,33], we cannot exclude that such sampling bias could lead to a significant underestimation of the dispersal capacity as measured by the WDC metric."

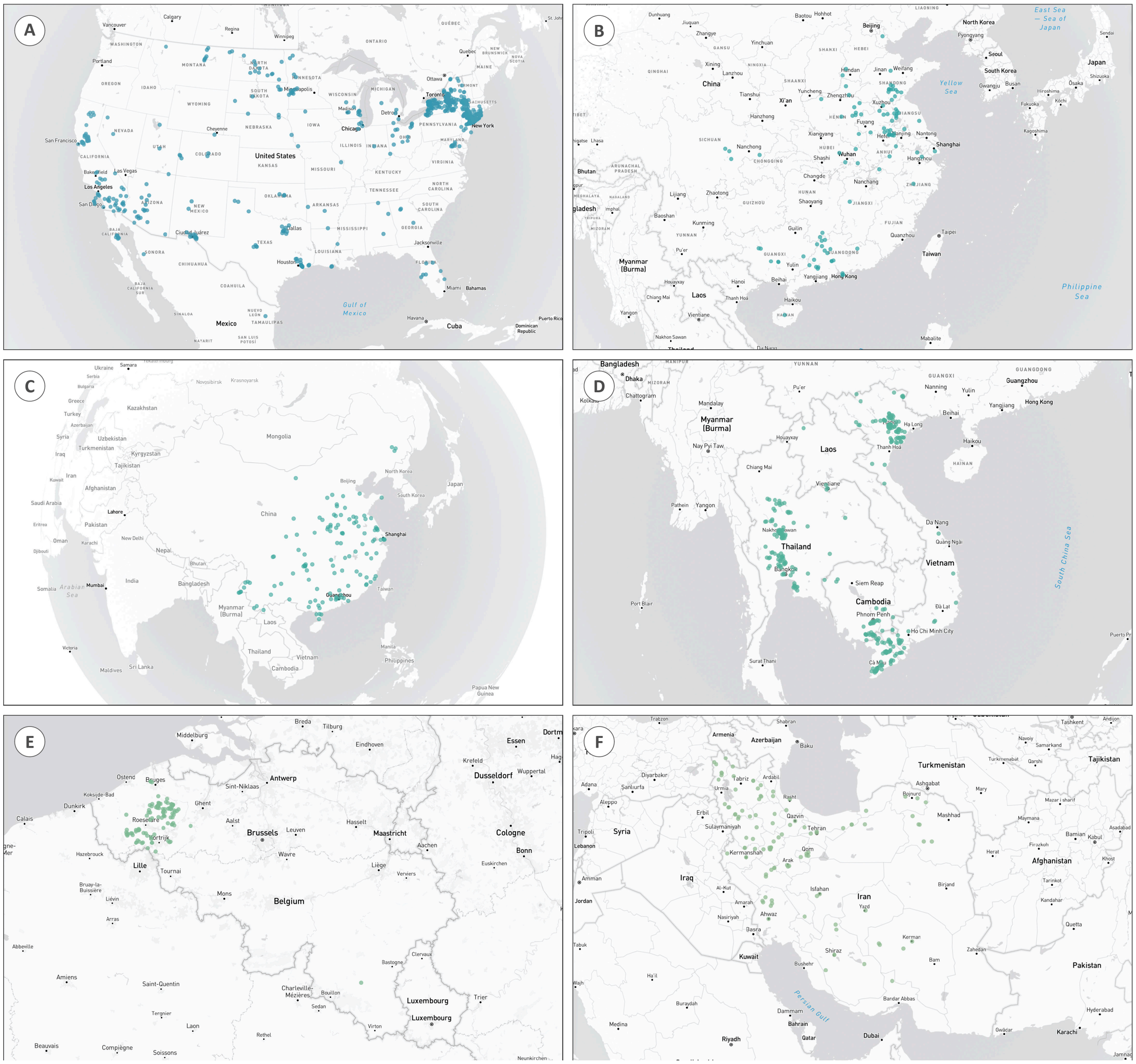

Figure S9 (part 1/3). sampling maps of the genomic sequences included in the empirical datasets analysed in the present study. *(**A**) West Nile virus, North America (Dellicour et al. 2020b); (**B**) Porcine deltacoronavirus, China (He et al. 2020); (**C**) Getah virus, China (Zhao et al. 2023); (**D**) AIV H5N1, Mekong region (Dellicour et al. 2020a); (**E**) AIV H3N1, Belgium (Van Borm et al. 2023a); (**F**) Rabies virus (dogs), Iran (Dellicour et al. 2019). [See Supplementary Information Figures for parts 2/3 and 3/3 of Figure S9]*

At some point it would be good to point out - if I'm right on this point - that WDC is not an unbiased estimator of the diffusion coefficient under the Brownian model (since locations of internal nodes in the phylogeny are estimated), and refer to something from phylogenetic comparative methods (a paper of Felsenstein, probably?) for a method that would.

Answer: We agree that the WDC is not in general an unbiased estimator of the diffusion coefficient under the Brownian model, since, as the Reviewer pointed out, it relies on the internal node locations, which are estimated. Its exact expectation will depend on the specific ancestral reconstruction method used, and has no reason to be exactly the diffusion coefficient. In the context of Bayesian phylogeography, such an estimator of the diffusion coefficient is obtained through a Bayesian analysis (typically relying on the Brownian or relaxed random walk models), which usually infer the whole diffusion variance matrix, with a specific coefficient for the latitude and longitude, and possibly a covariance term. The goal of the WDC metric is not to replace these estimators, but can be seen as a "realised" homogeneous diffusion coefficient that can be computed on any tree annotated with ancestral positions. We added a note to point this fact out, and referred to classical restricted maximum likelihood (Felsenstein 1973, PMID: 4741844) or Bayesian methods to infer the diffusion coefficient of a Brownian model (Lemey *et al*. 2010, PMID: 20203288) on a tree:

"Note that this relationship between the WDC and σ^2 is only valid in this toy model of a one-dimensional Brownian diffusion where the process is measured at all time steps dt. In a phylogenetic context, the geographic position of internal nodes of the tree is unknown, and the WDC is typically computed with inferred ancestral positions, so that the relationship between the WDC and σ^2 is not that simple in general and depends on the ancestral reconstruction method being used. The WDC metric should hence not be used as a replacement for classical estimators of the diffusion coefficient of a Brownian model, either based on the (restricted) maximum likelihood [31] or, in the context of this study, Bayesian inference [7]."

Abstract: "that can inform on" -> "that can be informative of" (or something)

Answer: Modification has been done.

Abstract: "recommendations for the *use of* lineage dispersal metrics"

Answer: Modification has been done.

Results: "can actually be expected" -> "is expected", perhaps?

Answer: Modification has been done.

Results: "quadratic distance" -> "squared distance"?

Answer: We prefer to keep "quadratic" to remain consistent with previously used terminology.

Results: "it seems that the random selection of sequences has often more impact on the dispersal metric estimates than the number of samples that were subsampled": My suspicion for why the different simulations in Figure S8 (i.e., the distinct draws from the RRW process) look so different is because you're using the Cauchy distribution, and the same plot for the Brownian model would look much more consistent between replicates. (Also see Conclusion.)

Answer: Thank you for pointing out this aspect. We agree that the use of a Cauchy distribution could also contribute to the observed variability among simulations, and we now explicitly mention it within the text: *"[...] As illustrated in Figure S8, our results indicate that when explicitly considering the uncertainty associated with the inference of the time-scaled tree topologies, the impact of various scenarios of sampling intensity on the dispersal metrics is less evident. Indeed, the posterior distribution of the dispersal metric estimates appears to be often quite large, sometimes preventing a clear distinction of any impact of sampling intensity on these estimates. Furthermore, in order to take into account the uncertainty related to phylogenetic inference, this additional simulation framework required performing a continuous phylogeographic inference based on each subset of geo-referenced tips sampled from the original simulation for generating various scenarios of sampling intensity (see the Materials and Methods section for further detail). Because of this additional inference step, it seems that the random selection of sequences has often more impact on the dispersal metric estimates than the number of samples that were subsampled, especially when the sampling size decreases (Fig. S8). The later result thus highlights the stochastic impacts of the sampling itself, independently of the impact of the sampling size. Finally, we also note that use of a RRW instead of a BRW diffusion model to conduct these continuous phylogeographic simulations could also contribute to the relatively large variability observed among simulations; a phenomenon that was already observed when comparing the results obtained when considering either a BRW or a RRW model to conduct the simulations under a birth-death process (Figs. 1, S3)."*

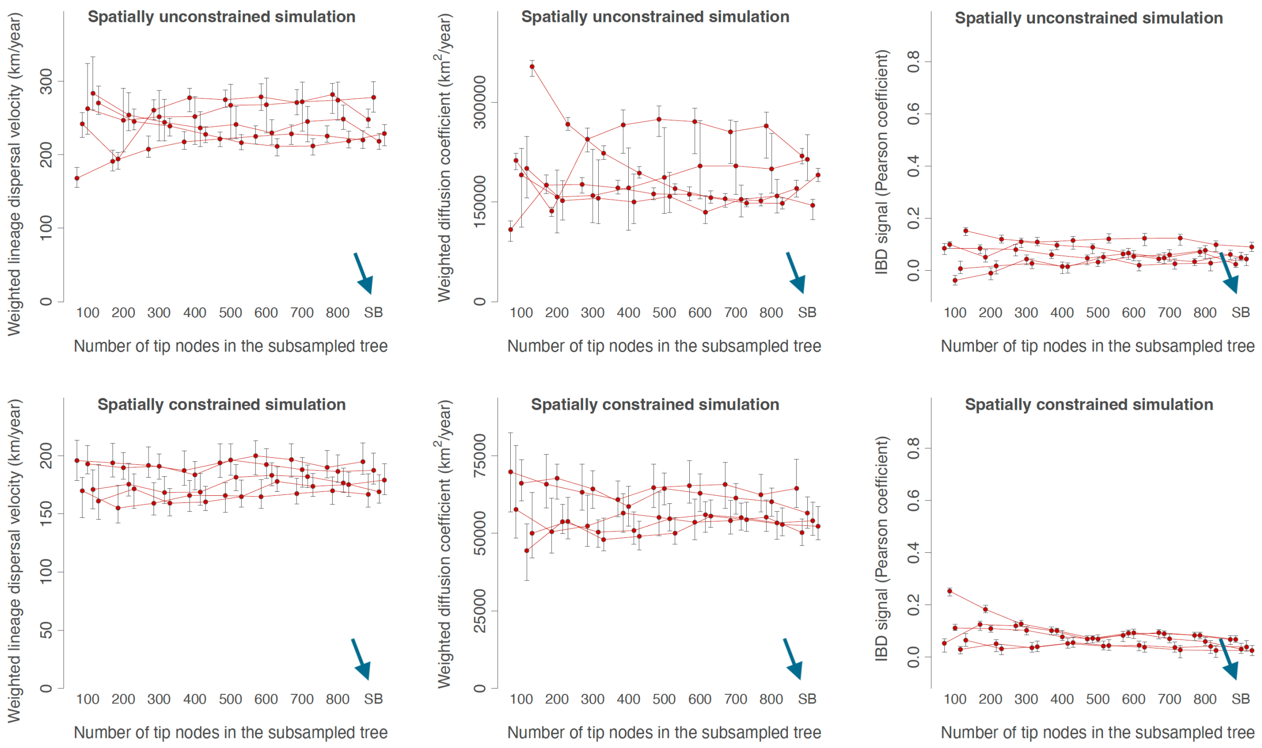

Figure S8 (modified): robustness of lineage dispersal metrics to the sampling effort and bias while incorporating the uncertainty associated with the phylogenetic inference. *[See the Figure in Supplementary information for the rest of the legend]. On this edited version of the figure, blue arrows highlight where the results associated with the scenario of sampling bias (SB) are reported.*

Figure S8: Where are the results for the "scenario of sampling bias (SB)"?

Answer: They are reported at the end of the x-axes of graphs included in Figure S8 (see below; highlighted with blue arrows), as well as in the following statements in the text, which have now been re-organised and expanded as a separate paragraph:

"As introduced above, we have also used these simulations of RRW diffusion processes along empirical tree branches to estimate the dispersal metrics from simulations based on a scenario of sampling bias where only a restricted area of the spread has been sampled (see the Materials and Methods section for further detail). Such a sampling bias scenario that has previously been shown to strongly affect continuous phylogeographic reconstructions [33]. Again, our results based on this simulation scenario and explicitly considering phylogenetic uncertainty do not indicate a clear impact of sampling bias on the estimates (Fig. S8). So with this simulation framework, we do not manage to distinguish a clear difference between the dispersal metrics estimated for the scenarios of various sampling intensity and sampling bias. The impact of such spatial sampling bias on the outcomes of continuous phylogeographic inference has been more thoroughly investigated in previous studies [8,33]. For instance, Kalkaukas and colleagues have explored the impact of three sampling bias scenarios on the estimation of the squared net lineage displacement per unit of time (σ^2, referred to as the "diffusion parameter" in their work [33]), which is directly related to a metric like WDC investigated here (see above). Their analyses reveal that all considered scenarios of sampling bias — a central sampling bias, a diagonal sampling bias, and a one-side sampling bias scenario similar to the one explored in this study — lead to an underestimation of the diffusion parameter. Further confirmed by additional investigations of sampling bias scenarios by Guindon & De Maio [8], these results thus indicate that sampling bias settings can in turn lead to an underestimation of the dispersal capacity as estimated by a metric like WDC."
