# Supplementary material for: How fast are viruses spreading in the wild?
Source: PLoS Biol. 2024 Dec 3;22(12):e3002914. doi: 10.1371/journal.pbio.3002914 (PMC11614233; doi:10.1371/journal.pbio.3002914)
Supplement: Attachment — Submitted filename: Response_to_Editor.pdf [file pbio.3002914.s004.pdf]

a) Please address my Data Policy requests below; specifically, we need you to supply the numerical values underlying Figs 1, 2, 3, S1, S2, S2, S3, S4, S5, S6, S7, S8, S9A-R, either as a supplementary data file or as a permanent DOI'd deposition. I note that you already have an associated GitHub deposition (https://eur01.safelinks.protection.outlook.com/?url=https%3A%2F%2Fgithub.com%2Fsdellicour%2Fdispersal_capacities&data=05%7C02%7Csimon.dellicour%40ulb.be%7Cf1e77922e58a440965be08dcf377180f%7C30a5145e75bd4212bb028ff9c0ea4ae9%7C0%7C0%7C638652940043441333%7CUnknown%7CTWFpbGZsb3d8eyJWIjoiMC4wLjAwMDAiLCJQIjoiV2luMzIiLCJBTiI6Ik1haWwiLCJXVCI6Mn0%3D%7C0%7C%7C%7C&sdata=0CNMKzFKWa6VJc4gZQOnH9dMNeliNGppyNaqIFeT1SI%3D&reserved=0); can you clarify that this contains the data underlying all the Figures?

Answer: We confirm that our GitHub repository contains the data underlying all the Figures included in our manuscript (Figures 1-3) and its Supplementary Information (Figures S1-S9).

Also, because Github depositions can be readily changed or deleted, please make a permanent DOI'd copy (e.g. in Zenodo) and provide this URL (see below).

Answer: We have now generated a permanent DOI (10.5281/zenodo.13984928) associated with our GitHub repo (https://doi.org/10.5281/zenodo.13984927).

b) Please cite the location of the data clearly in all relevant main and supplementary Figure legends, e.g. "The data underlying this Figure can be found in S1 Data" or "The data underlying this Figure can be found in https://eur01.safelinks.protection.outlook.com/?url=https%3A%2F%2Fzenodo.org%2Frecords%2FXXXXXXXX&data=05%7C02%7Csimon.dellicour%40ulb.be%7Cf1e77922e58a440965be08dcf377180f%7C30a5145e75bd4212bb028ff9c0ea4ae9%7C0%7C0%7C638652940043457439%7CUnknown%7CTWFpbGZsb3d8eyJWIjoiMC4wLjAwMDAiLCJQIjoiV2luMzIiLCJBTiI6Ik1haWwiLCJXVCI6Mn0%3D%7C0%7C%7C%7C&sdata=yOpbBZqbMeayhZhXvx5S%2BlBUWWUfVSh7R%2BOaY%2F5FKdY%3D&reserved=0

Answer: This has now been explicitly specified within each figure legend.

c) Please make any custom code available, either as a supplementary file or as part of your data deposition. I assume this will be in your Github/Zenodo deposition.

Answer: We confirm that this is already the case, as specified both in the section "Code and data availability" of the manuscript ("*R scripts related to the analyses based on simulated and real datasets are all available, along with the associated input/output files, at https://github.com/sdellicour/dispersal_capacities.*") and in the content of the "README" file displayed on the GitHub page of this public repository ("*R scripts related to the analyses based on simulated and real datasets are all available in All_R_scripts.r*").
